# Salting-out induced liquid–liquid microextraction for alogliptin benzoate determination in human plasma by HPLC/UV

**DOI:** 10.1186/s13065-020-00729-8

**Published:** 2021-01-15

**Authors:** Sherin F. Hammad, Inas A. Abdallah, Alaa Bedair, Fotouh R. Mansour

**Affiliations:** 1 Department of Pharmaceutical Analytical Chemistry, Faculty of Pharmacy, Tanta University, Elgeish Street, The Medical Campus of Tanta University, Tanta, 31111 Egypt; 2 Department of Analytical Chemistry, Faculty of Pharmacy, University of Sadat City, 32958 Sadat City, Egypt; 3 Pharmaceutical Services Center, Faculty of Pharmacy, Tanta University, Elgeish Street, The Medical Campus of Tanta University, Tanta, 31111 Egypt

**Keywords:** Alogliptin, Plasma, Salting-out, Microextraction, Sitagliptin

## Abstract

Salting-out induced liquid–liquid microextraction method has been developed for plasma sample treatment before determination of alogliptin by high performance liquid chromatography with UV detection. Several parameters were optimized to achieve maximum enrichment, including type of extractant, volume of extractant, type of anion, type of cation, salt amount and pH. The optimum conditions were attained using 500 µL of acetonitrile, added to 1 mL of aqueous sample containing 250 mg of sodium chloride at pH 12. An RP-HPLC method was developed and validated according to the International Conference on Harmonization guidelines M10. The method was linear in the concentration range of 0.1 to 50 µg/mL (correlation coefficient = 0.997). The limit of detection was 0.019 µg/mL and limit of quantitation was 0.06 µg/mL. The method was accurate and precise with an average % recovery of 99.7% and a % relative standard deviation ranging between 1.5 and 2.5. These results showed that the salting-out induced liquid–liquid microextraction methods could be better than other sample preparation protocols in terms of sensitivity, easiness, solvent consumption and waste reduction.

## Introduction

Sample preparation is a critical step in method development and application. The goal of sample preparation is to purify and concentrate the target analyte before analysis. Generally, sample treatment helps to enhance separation and quantitation of target analytes and protects analytical instruments [1]. Protein precipitation is the simplest biological sample preparation technique, as it requires simple mixing of samples with miscible organic solvents or mineral acids followed by centrifugation. However, the dilution effect of protein precipitation procedures compromises method sensitivity. Liquid–liquid extraction (LLE) is a better option for sample preparation that includes using large volumes of water immiscible organic solvents (e.g. Chloroform, ether, ethyl acetate), followed by evaporation and reconstitution in the least possible amount of a suitable solvent. These procedures render LLE labor-intensive, time-consuming and non-ecofriendly [2–4]. Moreover, LLE is limited to extraction of hydrophobic analytes due to the use of water immiscible hydrophobic extractants [5, 6].

Water-miscible polar organic solvents can be used for sample preparation in a mode known as salting-out induced liquid–liquid extraction (SALLE). In SALLE, solvents such as acetonitrile, isopropanol and acetone are mixed with the aqueous sample before phase separation is induced by adding enough amount of a suitable salt [7]. SALLE is an efficient technique for extraction of hydrophilic analytes [8, 9] and the employment of water miscible organic solvents make SALLE compatible with most analytical methods. SALLE has already been used for extraction of drugs from different matrices, including plasma [10–13] whole blood [14], serum[15], urine [16], fruit juice [17], milk [18] and water [19, 20].

Miniaturization of sample preparation techniques is a recent trend in analytical chemistry. Decreasing solvent consumption decreases the cost of analysis, reduces organic wastes, protect operator’s health and preserve the environment. Miniaturized liquid–liquid extraction (also known as liquid–liquid microextraction, LLME) differs from conventional LLE in two aspects [21, 22]: the need for microliters of organic solvents rather than milliliters, and the tendency to extract small but representative amounts of the analyte in the aqueous sample. This small extracted amount of the drug will be highly concentrated in the minute volume of organic extractant, which makes LLME highly efficient in sample pre-concentration.

**Fig. 1:**
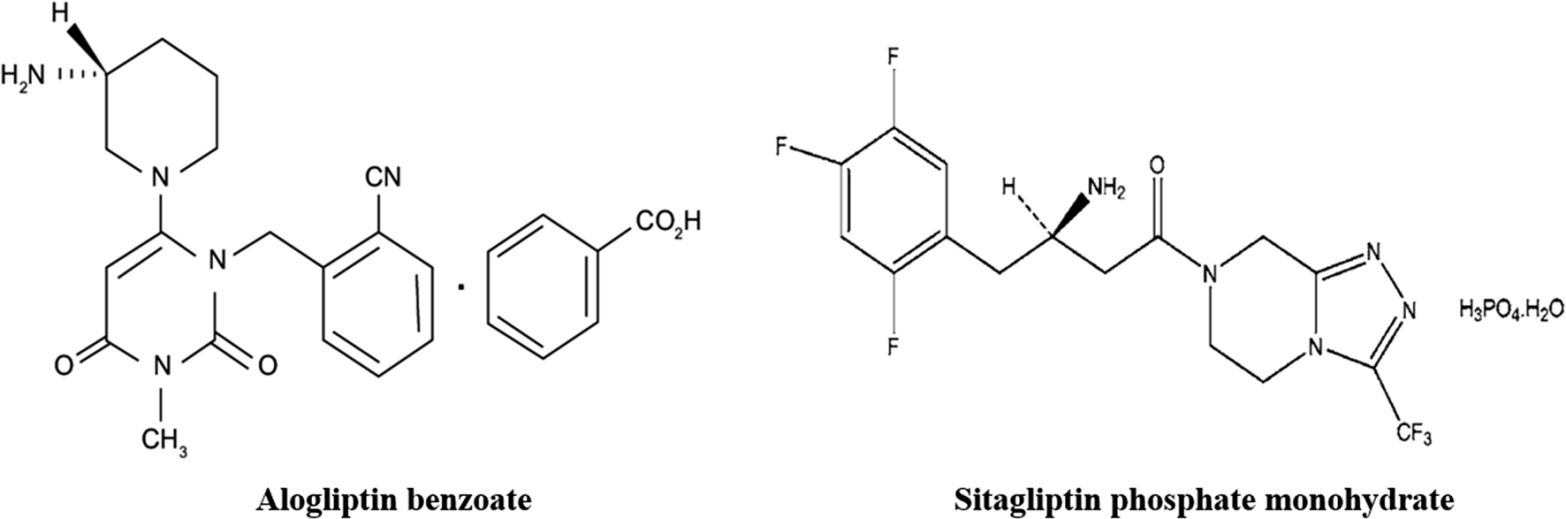
The chemical structure of the analyte (alogliptin benzoate) and the internal standard (sitagliptin phosphate monohydrate)

In this work, we present salting-out induced liquid-liquid microextraction (SALLME) for preparation and analysis of alogliptin in human plasma by HPLC/UV, using sitagliptin as an internal standard (IS). Figure 1 shows the chemical structures of the drug and the internal standard. Alogliptin is an oral drug for Type 2 diabetes mellitus that works by inhibiting dipeptidyl peptidase 4 (DPP-4). The reported C_max_ of alogliptin was 0.11 µg/mL at a dose of 25 mg [23]. Due to the low C_max_, determination of alogliptin in plasma necessitated using highly sensitive techniques such as LC–MS/MS after protein precipitation of samples [23–26]. However, protein precipitation induces ionization suppression in MS detection [27, 28] and precludes detection by other less sensitive detectors such as UV. Using SALLME in sample preparation enabled enrichment and determination of alogliptin in plasma using HPLC/UV with acceptable accuracy, precision and sensitivity.

## Experimental

### Chemicals and reagents

Alogliptin benzoate (99.7%) and sitagliptin phosphate monohydrate (99.8%) were kindly supplied from Global Nabi Pharmaceuticals (6th of October City, Egypt). Acetonitrile, potassium dihydrogen phosphate, phosphoric acid, methanol and sodium hydroxide were purchased from Merck (Darmstadt, Germany). Sodium carbonate and sodium sulfate were purchased from Pharaohs Chem (Obour City, Egypt). Sodium chloride and propylene glycol were purchased from El-Gomhouria Company (Cairo, Egypt). Sodium acetate, sodium thiosulfate and sodium dihydrogen phosphate were purchased from Alpha Chemicals (Cairo, Egypt). Glycerol was purchased from Sigma Aldrich (St. Louis, MO, USA). Tetrahydrofuran was purchased from Universal Fine Chemicals (Sanborn, NY, USA) while calcium chloride and potassium chloride were purchased from Piochem (6th of October, Egypt). Magnesium chloride was purchased from Chem Lab (Zedelgem, Belgium). Human plasma samples were kindly provided by Vacsera National Blood Bank, (Giza, Egypt)

### Instrumentation

The determination of alogliptin was done on a Dionex UltiMate 3000 HPLC (Thermo Scientific™, Dionex™, Sunnyvale, CA, USA). The instrument composed of a WPS-3000TSL autosampler, an LPG-3400SD quaternary pump, a VWD-3000 variable wavelength detector and a TCC-3000SD column thermostat. Data processing and acquisition were carried out by Chromeleon 7 software. Tabletop Cyan-CL008 centrifuge (Hulshout,Belgium) was used. The pH values were adjusted by Jenway 3510 pH-meter (Staffordshire,UK).

### Chromatographic conditions

The chromatographic conditions were run in an isocratic elution mode, using a mobile phase consisting of 50 mM phosphate buffer (pH = 2.5) and acetonitrile in a ratio of 70: 30, v/v. The volume of injection was 5 µL, the flow rate was 1 mL/min, the wavelength of detection was 210 nm and the column temperature was set at 30 °C (303K) .Chromatographic separation was done on a Thermo Hypersil ODS C18 column (150 mm × 4.6 mm, 5 µm).

### Stock and working solutions

Stock solutions of alogliptin benzoate and sitagliptin phosphate monohydrate (1 mg/mL for each) were separately prepared in distilled water, and stored at 4 °C (277 K). To study different conditions on extraction performance, water samples were obtained by spiking distilled water with alogliptin at a concentration of 25 µg/mL.

### Extraction procedure

**Fig. 2:**
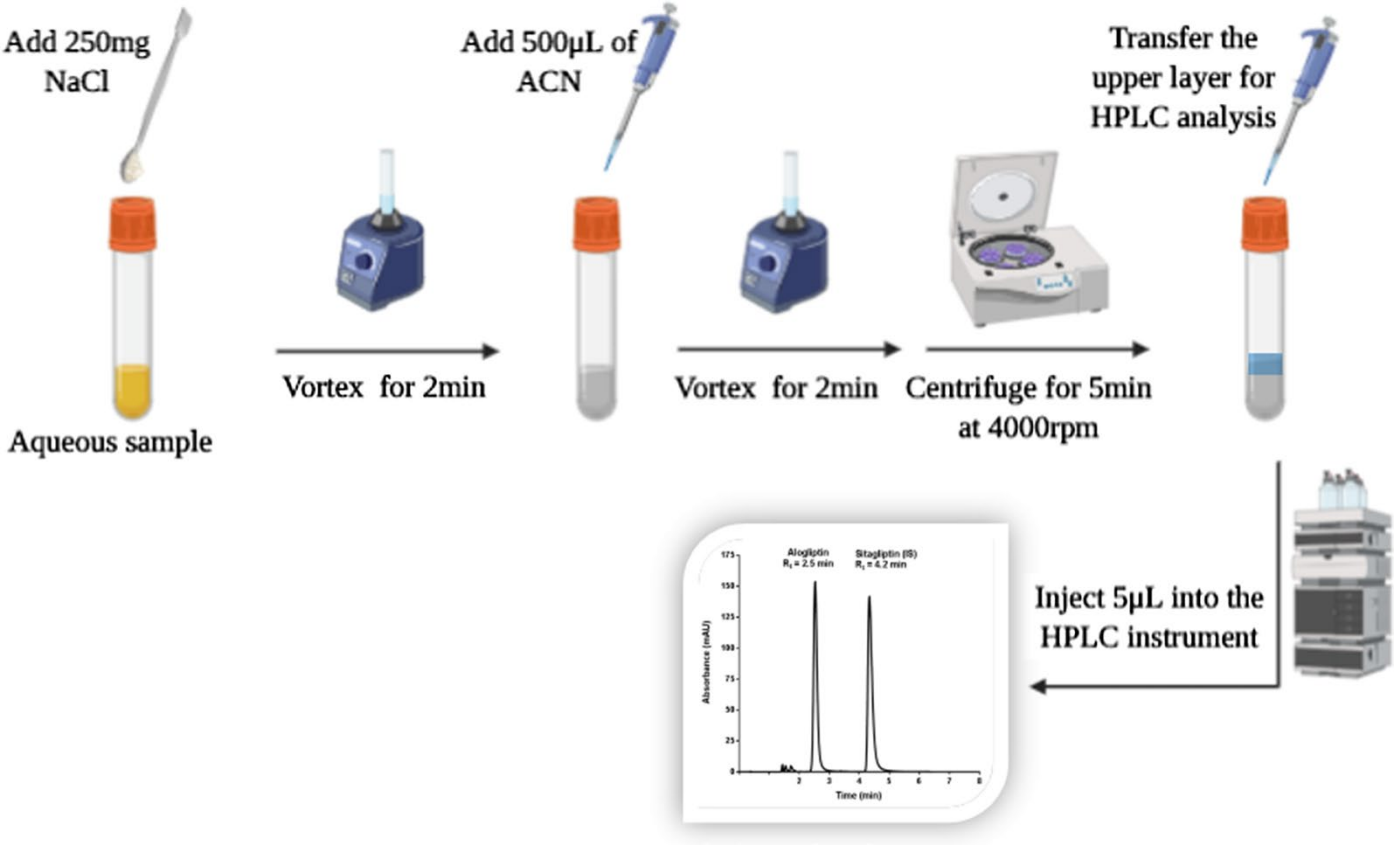
Procedures of salting-out induced liquid–liquid microextraction for alogliptin benzoate from aqueous samples using sitagliptin phosphate monohydrate as an internal standard

The procedures of SALLME were optimized as follows: salt amount (Sodium chloride) (250 mg) was added to 5 mL screw cap glass test tube containing 1 mL of aqueous sample solution then the tube was vortexed for 2 min. A volume of 500 µL of acetonitrile was added to the previous solution, then the tube was vortexed again for 2 min followed by centrifugation for 5 min at 4000 rpm. The upper layer was pipetted (20 µL) and was transferred into an HPLC vial for analysis. Figure 2 summarizes the applied SALLME procedures for alogliptin.

### Method performance evaluation

The validation of SALLME was performed by spiking of plasma with alogliptin benzoate and using sitagliptin phosphate monohydrate as internal standard. The therapeutic level of alogliptin was used as a reference in selecting the linearity range [23]. The calibration curve was constructed by plotting the analyte-to-IS peak area ratio (*y*) versus the analyte nominal concentration (*x*). The method sensitivity was determined by the limit of detection (LOD) and limit of quantitation (LOQ). The linearity of the method was assessed by calculating the correlation coefficient. Intra-day (n = 3) and inter-day (n = 3) accuracy and precision determined by % recovery and relative standard deviation (Table 2).

### Freeze and thaw stability study

The freeze and thaw stability of alogliptin was investigated in human plasma at three concentration levels: 5, 20, 40 µg/mL. The plasma samples were spiked with the quality control concentrations and frozen at − 20 °C (253 K), and the samples were later thawed at room temperature till complete thawing, then analyzed. This process of freeze and thaw was repeated for three cycles, and the alogliptin concentration was measured after each cycle and compared with the zero cycle concentration. The samples were considered stable if the % recovery were within 15% of the nominal concentrations and % relative standard deviation (RSD) were less than 15% [29].

### Application to biological samples

Human blood Plasma (400 µL) was spiked with different concentrations of alogliptin and the internal standard (25 µg/mL) to attain the desired therapeutic levels of the drug and to construct the calibration curve. Then, 500 µL of acetonitrile was added to plasma, vortexed for 2 min, then the tube was centrifuged for 5 min at 4000 rpm. The upper layer was transferred to another test tube followed by the addition of 10 µL of 1 M NaOH to adjust the pH value at 12. Then 490 µL of distilled water and 250 mg sodium chloride were added and the tube was vortexed for 2 min followed by centrifugation for 5 min at 4000 rpm. The upper layer was pipetted and transferred to HPLC vial for analysis.

### Statistical analysis

The optimization parameters were studied using three replicates and the data were presented as the average ± standard deviation (n = 3). Statistical analysis was performed using the SPSS statistical package (V. 24) to compare the mean and the variance of the developed method with the reference method using Student t-test and F-test. The results of the comparison study were expressed as average ± standard deviation (n = 3), and the p-values were calculated for both tests at the 95% confidence level (α = 0.05).

## Result and discussion

In this work, SALLME was tried for alogliptin extraction from plasma. Different factors were studied to achieve the maximum enrichment. Different solvents, volumes of extractant, anions, cations, amounts of salt and pH values were investigated. Optimization of these parameters was performed using one-factor-at-a-time (OFAT). The peak area was the parameter to evaluate the effect of each factor on extraction.

### Organic solvent optimization

Preliminary experiments were done to investigate the optimum extractant using different solvents including methanol, propylene glycol, glycerol, tetrahydrofuran and acetonitrile. A 2000 µL of each solvent was added to 1 mL of aqueous solution containing 250 mg of sodium chloride followed by vortexing for 2 min, then the tube was centrifuged for 5 min at 4000 rpm. The results showed that there is no phase separation with methanol, propylene glycol, and glycerol, which could be due to the high polarity of the hydroxyl groups and the multiple H-bonds formed between these solvents and water. On the other hand, both tetrahydrofuran and acetonitrile could induce phase separation, but the background noise in tetrahydrofuran after injection into HPLC/UV was significantly higher than acetonitrile. For this reason, acetonitrile was chosen as the optimum extractant in the following SALLME procedures.

### Acetonitrile volume optimization

**Fig. 3:**
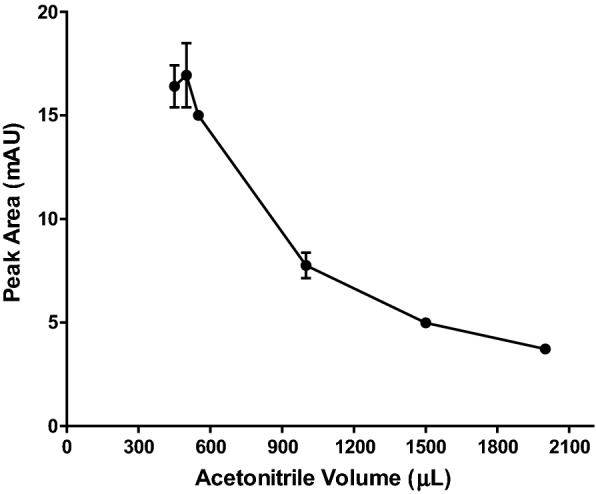
Effect of acetonitrile volume (µL) on the efficiency of SALLME of alogliptin benzoate

The extractant volume is the most important factor that could affect the sample enrichment in SALLME. Generally, analyte pre-concentration is inversely proportional to the volume of extractant [30]. Different volumes of acetonitrile were investigated in the range of 100 to 2000 µL. The results showed that 450 µL was the least volume of acetonitrile that could be used in SALLME. Using volumes of acetonitrile lower than 450 µL could not induce phase separation. As shown in Fig. 3, the highest response was observed using 500 µL acetonitrile, thus it was designated as the optimum acetonitrile volume in the following procedures.

### Anion- type optimization

**Fig. 4:**
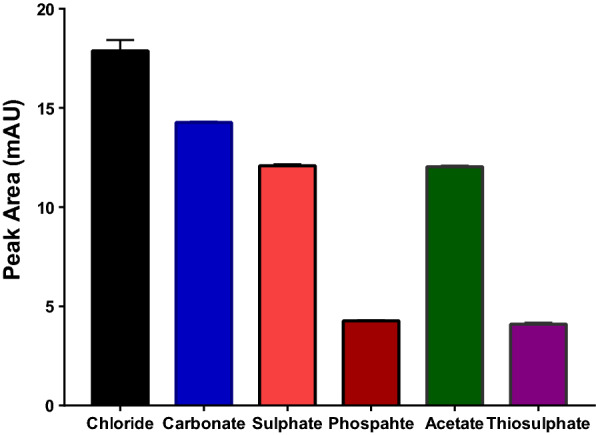
Effect of extractant type on the efficiency of SALLME of alogliptin benzoate

Different anions were investigated to select the optimum anion that could achieve the best extraction efficiency. All anions were sodium salts of monovalent (Chloride and acetate), divalent (Sulfate, thiosulfate, carbonate) and trivalent (Phosphate) anions. Figure 4 shows that the highest extraction efficiency was achieved with chloride. The mechanism of salting-out depends on hydrophobic effect and electrostatic repulsion [31]. In this aspect, ions with high charge density (charge/size) are expected to interact strongly with water and induce more electrostatic repulsion [32]. The small size of chloride compared with other anions may explain the observed high efficiency. It is here worth mentioning that the volume retrieved of acetonitrile after adding chloride was small compared with other anions which helped make the analyte more concentrated in the separated layer of acetonitrile. Further optimization was performed by using a binary mixture of chloride/carbonate, the two anions that could attain the best results, but in different ratios. Adding carbonate to chloride resulted in higher phase ratios (Retrieved volume of ACN/Aqueous volume). However, using chloride alone was better than its mixtures with carbonate. Thus, chloride was selected as the optimum anion in this step.

### Cation-type optimization

The type of salt cation has a role in salting-out phenomena, but to less extent than the anion component. Seven cations were tested, all in chloride salts including monovalent (Soidum and potassium), divalent (Copper, cobalt, calcium and magnesium) and trivalent (Ferric) cations. Transition metals (Copper, cobalt and ferric) were found not suitable due to the observed color, which could complicate the sample matrix. No phase separation was observed in case of potassium and magnesium, while sodium and calcium could successfully induce salting out of the acetonitrile layer. Compared with calcium, sodium could achieve higher extraction efficiency due to the small size of sodium, which could enhance charge density and salting out capabilities [31]. Therefore, sodium chloride was selected as an optimum salt for SALLME of alogliptin from plasma, because it was cheap, safe, available and more efficient.

### pH Optimization

**Fig. 5:**
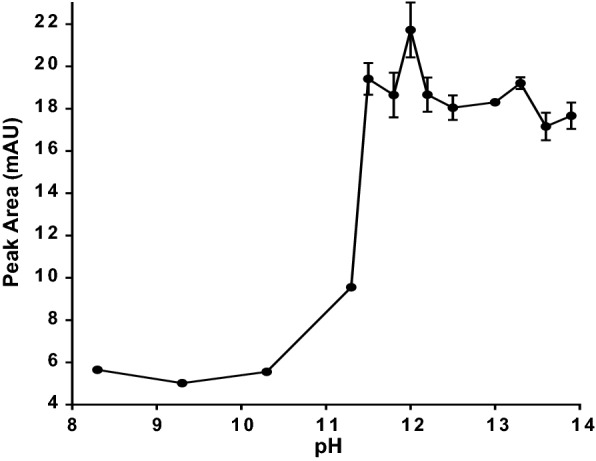
Effect of diluent pH on the efficiency of SALLME of alogliptin benzoate

In extraction methods, pH plays a major role due to its effect on solubility and ionization of drugs. Different pH values were investigated in the range of 8.3 to 13.9, adjusted using the appropriate concentration of sodium hydroxide to span the pKa value of alogliptin (pKa = 9.47). Figure 5 shows low peak areas for alogliptin at pH values lower than 9.47 due to the predominance of the ionized form. Further increases in pH were associated with a corresponding pronounced increase in response up to pH 11.5. Increasing pH above 11.5 did not significantly affect the obtained response. The highest extraction efficiency was achieved with pH = 12 followed by a steady state in the range of 13 to 13.9. At pH = 12, alogliptin will be in the non-ionized form, leading to better extraction efficiency. Therefore, the selected optimum pH value was 12.

### Chloride amount optimization

**Fig. 6:**
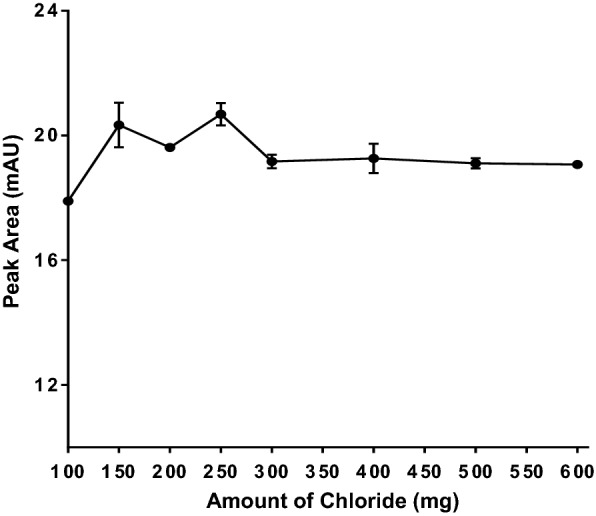
Effect of chloride amount (in mg) on the efficiency of SALLME of alogliptin benzoate

To study the effect of salt amount on SALLME performance, different amounts of sodium chloride were tried in the range of 100 mg to 600 mg. The results showed that the salt amount had a small effect on extraction efficiency. The peak areas of alogliptin benzoate were comparable regardless of the amount of sodium chloride. As shown in Fig. 6, 250 mg of NaCl resulted in slightly higher responses, thus it was selected as the optimum amount in the following procedures.

### Method validation

The chromatographic method was validated according to the International Conference of Harmonization (ICH) guidelines M10 [29]. The following parameters were investigated: linearity, precision, accuracy, limit of detection (LOD) and limit of quantitation (LOQ). The method was found linear in the range of 0.1 to 50 µg/mL Accuracy and precision were determined by % recovery and % RSD, respectively. The results were satisfactory for bioanalysis application.

### Linearity

The method linearity was investigated in the concentration range of 0.1 to 50 µg/mL. The calibration curve was constructed by plotting the response ratio (ratio between peak area of alogliptin and peak area of sitagliptin) on the y-axis and alogliptin concentration on the x-axis (in µg/mL). The calibration curve indicated a linear relationship between response ratio and alogliptin concentration with an acceptable correlation coefficient and regression parameters as summarized in Table 1.

**Table 1:** Quantitative analysis and regression line

Compound	Slope	Intercept	r	Range	LOD	LOQ
Alogliptin	0.0272	0.0012	0.997	0.1–50 µg/mL	0.019 µg/mL	0.06 µg/mL

*R*^2^ Regression coefficient, *LOD* Limit of detection, *LOQ* Limit of quantitation

### Accuracy and precision

Method accuracy was investigated by analyzing plasma samples, spiked with alogliptin at three concentrations: 5, 20 and 40 µg/mL, each prepared in triplicate. Table 2 indicates that the % recovery was in the range of 98 to 101, with an average %recovery of 99.76. The precision of the analytical method was investigated by analyzing plasma samples, spiked with alogliptin at the same three concentrations, each prepared in triplicate. The concentrations were analyzed in the same day to test repeatability and in 3 consecutive days to evaluate intermediate precision. Based on the %RSD shown in Table 2, the method intraday and interday precision were acceptable for bioanalysis application.

**Table 2:** Intra-day and inter-day precision and accuracy

Parameter	Accuracy & precision					
	Intraday			Interday		
	Added (µg/mL)	Found (µg/mL)	Found (%)	Added (µg/mL)	Found (µg/mL)	Found (%)
Alogliptin	5	5.05	101.00	5	5.02	100.44
	20	19.97	99.85	20	19.99	99.93
	40	39.38	98.43	40	39.60	99.01
Mean			99.76			99.79
% RSD			1.56			2.57

*RSD* relative standard deviation

### Limit of detection and Limit of quantitation

Limit of detection (LOD) and limit of quantitation (LOQ) were calculated according to the following equations:

$$LOD\,=\,\frac{3.3\,\times\,S.D\,of\,Blank}{Calibration\,curve\,slope}$$

$$LOQ= \frac{10\,\times\,S.D\,of\,Blank}{Calibration\,curve slope}$$

where SD is the standard deviation of 10 blank injections. LOD was 0.019 µg/mL and LOQ was 0.06 µg/mL. SALLME was found sensitive for determination alogliptin in biological samples obtained from alogliptin clinical study.

### Freeze and thaw stability study

The freeze and thaw stability study was performed by spiking human plasma with alogliptin at three quality control concentration levels (5, 20 and 40 µg/mL). The plasma samples were frozen for 12 hours, thawed for three cycles, and the concentration of alogliptin was determined after each cycle to be compared with the zero cycle. Table 3 indicates that alogliptin was stable for three freeze/thaw cycles.

**Table 3:** Freeze and thaw stability study

	Added (µg/mL)	Found (µg/mL)	Recovery (%)	RSD (%)	RE (%)
1st cycle	5.00	4.98	99.63	4.28	0.37
	20.00	19.98	99.91	3.39	0.09
	40.00	41.03	102.58	3.26	2.58
2nd cycle	5.00	5.21	104.23	1.94	4.23
	20.00	19.88	99.38	1.54	0.62
	40.00	39.34	98.36	1.49	1.64
3rd cycle	5.00	5.09	101.73	3.66	1.73
	20.00	20.57	102.83	0.98	2.83
	40.00	38.89	97.23	4.92	2.77

### Comparison with other reported methods

**Fig. 7:**
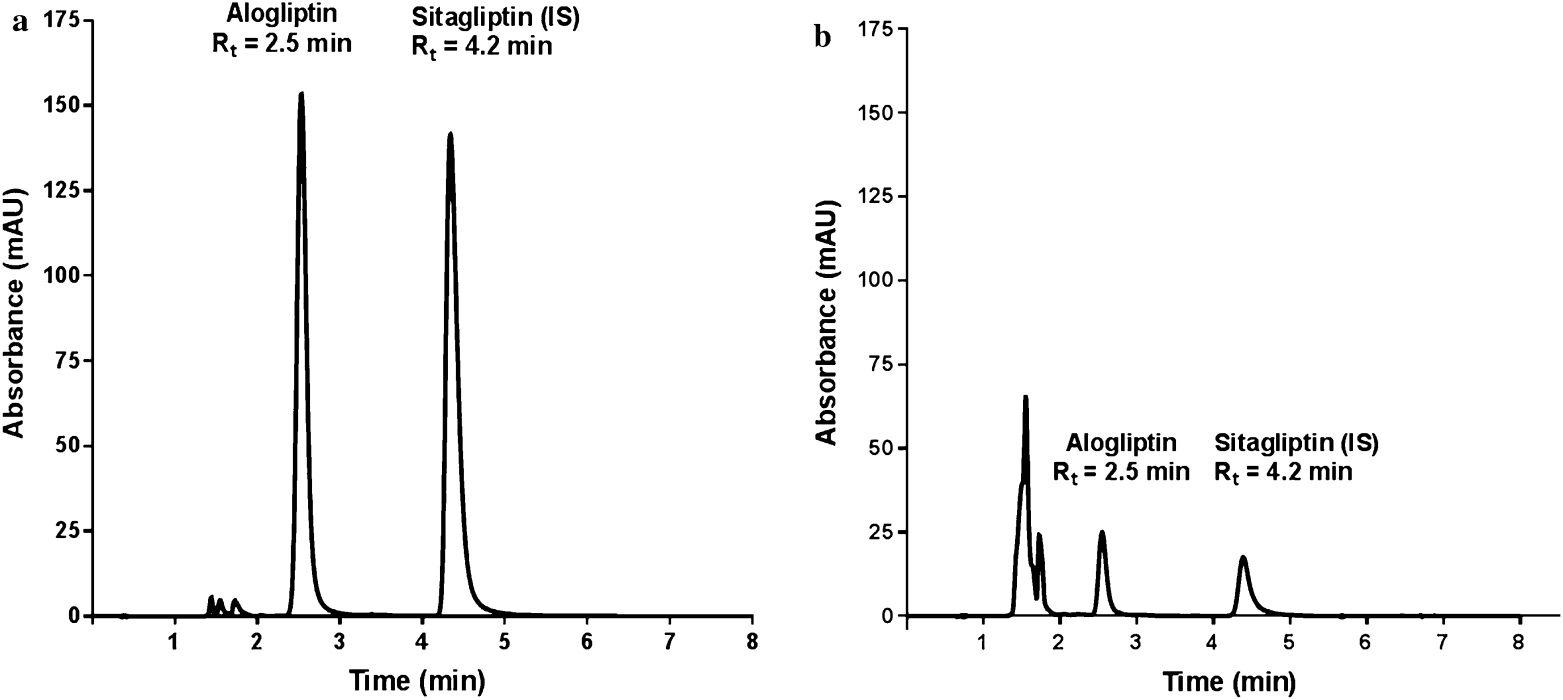
Chromatographic separation of alogliptin benzoate and sitagliptin phosphate monohydrate using the proposed SALLME method (**a**) and the reported protein precipitation method (**b**)

Due to the low plasma concentrations of alogliptin, most analytical methods used LC-MS/MS for quantitation after sample preparation using protein precipitation. While protein precipitation is a simple and fast method for sample treatment, its efficiency to remove interference and protect analytical instruments is less than perfect. Moreover, the dilution effect of the added precipitating agent compromises method sensitivity, which could be compensated by the inherent high sensitivity of mass detection. For sake of comparison, the developed SALLME method was compared with the reported protein precipitation procedure [27] under the same chromatographic conditions. As shown in Fig. 7, a huge plasma peak appeared in the beginning of the chromatogram (retention time = 1.55 min) compared with a very small peak at the same retention time in SALLME. This could be due to the dual function of acetonitrile to precipitate protein and to extract the drug after salting out. Moreover, the peak area of alogliptin treated with SALLME was more than seven times higher than that in the protein precipitation method. These results were achieved while only one third of the acetonitrile volume was consumed in the SALLME method which decreases organic solvent consumption, reduces organic waste, protects the operator and the environment and bestows green characteristics on the developed method.

Besides protein precipitation, conventional LLE [33] was also used for alogliptin determination in plasma by UPLC/DAD using diethyl ether as an extractant and a mobile phase consisting of acetonitrile:phosphate buffer (50:50, v/v) for reconstitution after evaporation. The results of the developed SALLME method were compared with the results of the reported LLE method using Student t-test and F-test at 95% confidence level (α = 0.05) as Table 4 shows. The statistical analysis indicated that there were no significant differences between the two methods. Although these results showed comparable accuracy and precision, the procedures in LLE were time consuming, non-ecofriendly, more expensive and required vacuum for solvent evaporation. On the other side, SALLME was simpler, cheaper, eco-friendlier and could achieve lower LOQ than the reported LLE method.

**Table 4:** Comparison between the developed SALLME method and the reference LLE method [33]

Parameter	Comparison with reference method					
	The SALLME method			Reference method		
	Added (µg/mL)	Found* (µg/mL)	Found (%)	Added (µg/mL)	Found (µg/mL)	Found (%)
	5.00	4.96	99.15	5.00	4.96	99.29
	10.00	10.08	100.75	10.00	10.23	102.26
	20.00	20.02	100.08	20.00	20.20	101.01
Mean			99.99			100.85
% RSD			0.81			1.48
t-test**			0.429			
F-test**			0.450			

^* ^Average of five determinations for each concentration

** p-values at 95% confidence level (α = 0.05)

## Conclusions

A SALLME method was developed for preparation of plasma samples for HPLC analysis. The method provided a simple, economical, fast and green approach for alogliptin extraction from biological samples. Moreover, acetonitrile, the employed extractant is compatible with different analytical instruments and HPLC detectors. The ability of this sample treatment to pre-concentrate the sample makes possible UV detection after chromatographic separation under isocratic conditions. SALLME can be applied to other pharmaceutical compounds, especially highly polar drugs that are difficult to extract using conventional extracting solvents such as chloroform, methylene chloride, ethyl acetate and ether. Future work includes applying SALLME as a preparation method for other drugs using other analytical techniques such as UV/Vis spectroscopy, spectrofluorometry and capillary electrophoresis. Applying similar procedures for extraction of macromolecules is also in our scope. SALLME paves the way for simpler and greener extraction methods using the least possible amount of organic solvents. The small volumes and the safety of the organic solvents employed in SALLME makes it appreciably greener compared with the high volumes and the health hazardousness of other solvents used in conventional extraction.

## Data Availability

The datasets used during the current study are available from the corresponding author on reasonable request.

## Abbreviations

DAD: Diode-array detector
DPP-4: Dipeptidyl peptidase 4
ICH: International Conference of Harmonization
IS: Internal standard
LLE: Liquid–liquid extraction
LLME: Liquid–liquid microextraction
LOD: Limit of detection
LOQ: Limit of quantitation
OFAT: One-factor-at-a-time
RE: Relative error
RSD: Relative standard deviation
SALLE: Salting-out induced liquid–liquid extraction
SALLME: Salting-out induced liquid–liquid microextraction
